# A maxillary location of extra-nodal Rosai Dorfman disease: a case report

**DOI:** 10.11604/pamj.2024.48.20.43457

**Published:** 2024-05-24

**Authors:** Yassine Bennaoui, Mohamed Salah Koussay Hattab, Fadoua Fettal, Zakaria Aziz, Hanane Rais, Nadia Mansouri Hattab

**Affiliations:** 1 Maxillo Facial Surgery Department, University Hospital Center Mohammed VI, Marrakech, Morocco

**Keywords:** Rosai-Dorfman, sinus histiocytosis, emperipolesis, maxilla, case report

## Abstract

**Introduction:** Rosai-Dorfman disease (RDD) is a rare benign histiocytic proliferation, characterized by a group of clinical symptoms. This report presents a case of extranodal RDD manifesting as a progressively enlarging left maxillary mass in a 42-year-old woman. Surgical exploration and biopsy confirmed the diagnosis of RDD, with characteristic histopathological features including emperipolesis. Treatment involved corticotherapy, resulting in controlled maxillary pain and improvement of the disease after one year. This case underscores the potential for extra-nodal RDD presentations, posing diagnostic challenges and emphasizing the importance of considering RDD in the differential diagnosis of maxillary masses.

## Introduction

Rosai-Dorfman disease (RDD), also known as sinus histiocytosis with massive lymphadenopathy (SHML), is a rare non-Langerhans cell histiocytosis characterized by the overproduction and accumulation of histiocytes, a type of immune cell, in various tissues of the body. First described by pathologists Juan Rosai and Ronald Dorfman in 1969, RDD primarily affects the lymph nodes but can also involve other organs. It primarily affects children and young adults. The most common clinical sign is the presence of massive cervical lymphadenopathy (90% of cases). often accompanied by facial swelling. In rare cases, RDD can affect the central nervous system, leading to neurological symptoms. Extranodal locations, such as the nasal cavity and paranasal sinuses, are reported in 11% of cases. Diagnosis can be challenging due to the rarity of the disease and similarities in symptoms with other conditions like lymphoma or tuberculosis. However, a biopsy of the affected tissue confirms the diagnosis by revealing characteristic histiocytic cells. Treatment options depend on the severity and location of the disease. ranging from observation to interventions such as corticosteroids, chemotherapy, radiotherapy or surgery. Overall, the prognosis for RDD is generally good, with most patients experiencing complete remission. In rare cases, the disease may become chronic, requiring ongoing treatment [1,2].

## Patient and observation

**Figure 1:**
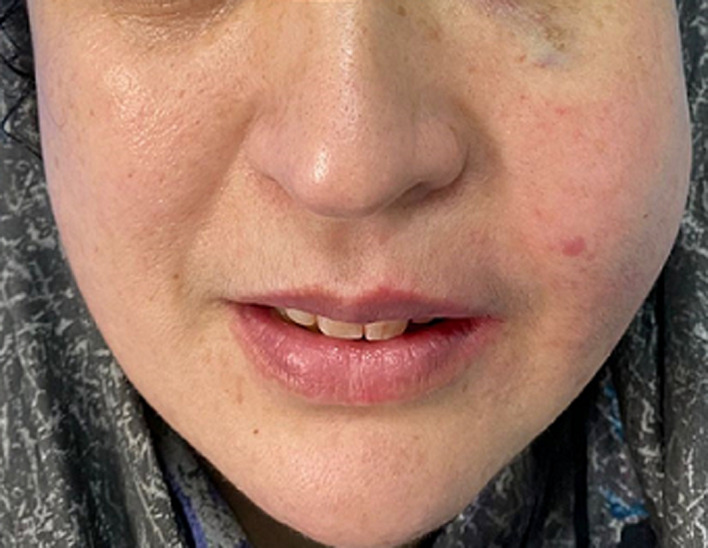
left maxillary swelling in Rosai-Dorfman case

**Patient information:** we report a case of a 42-year-old woman, without a significant medical history, who was referred to our institution because of a progressively growing left cheek swelling over the past year (Figure 1).

**Clinical findings:** physical examination revealed a firm, painless, and poorly defined left cheek swelling. Facial sensitivity and ocular mobility is preserved without palpable cervical lymphadenopathy. On intraoral examination, a left underlying mass appeared to bulge and distort the gingivobuccal sulcus without dental mobility with a normal mouth opening.

**Timeline of current episode:** in March 2022: clinical assessment and imaging, April 2022: biopsy and immunohistochemically study were conducted June 2023: beginning of treatment, December 2023: disappearance of the maxillary mass after one year of corticotherapy.

**Figure 2:**
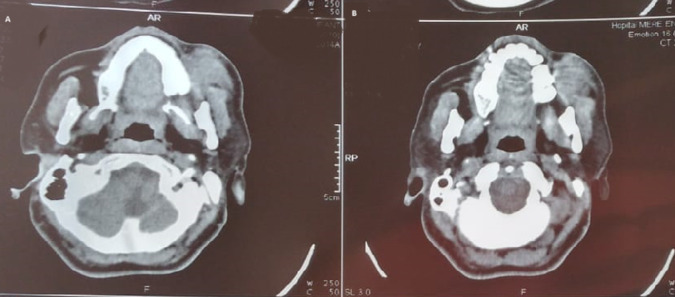
computed tomography scan revealing left maxillary mass with osteolytic features in Rosai-Dorfman disease

**Figure 3:**
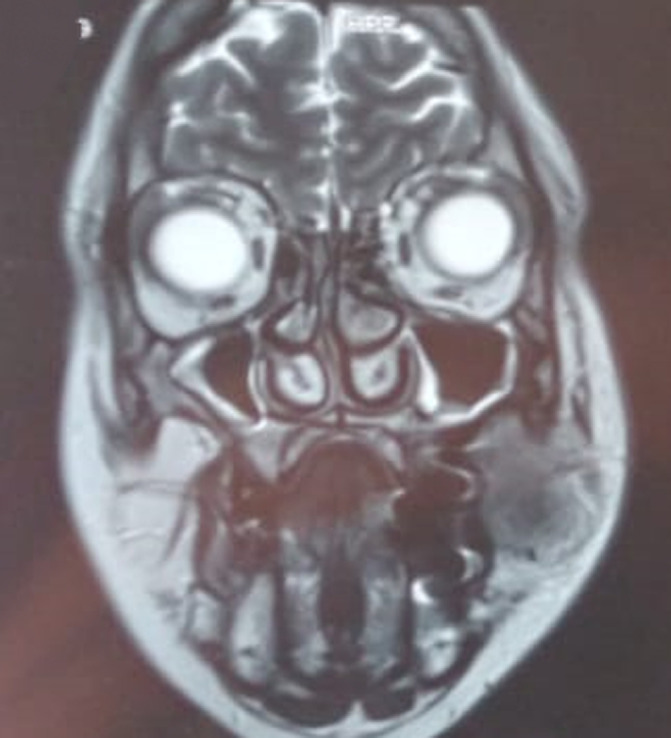
magnetic resonance imaging demonstrating osteolysis and masseter muscle infiltration in Rosai-Dorfman case

**Figure 4:**
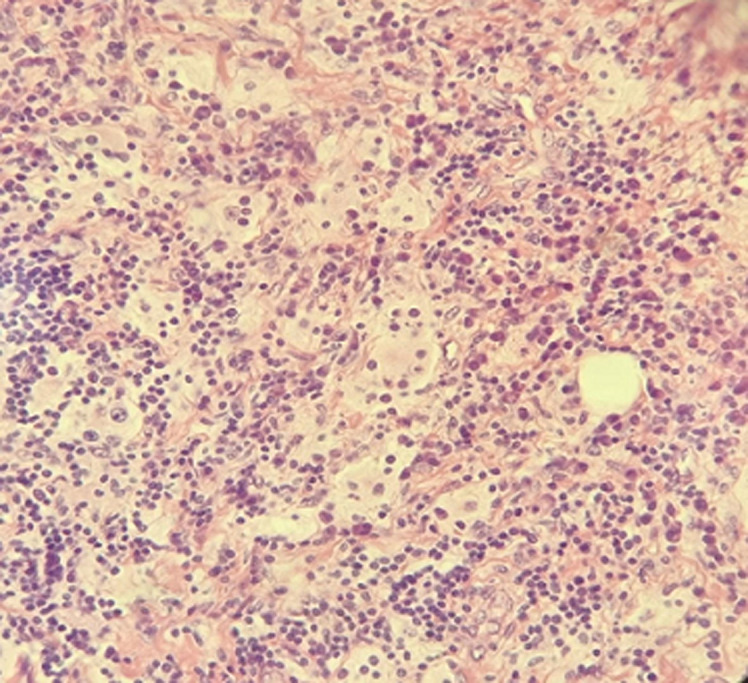
histological examination showing an emperipolesis

**Diagnostic assessment:** further evaluation included facial computed tomography (CT) and magnetic resonance imaging (MRI), showing a mass measuring approximately 34 x 28 x 32 mm in the left cheek, with osteolytic image of the maxillary bone. Outside, it comes into contact with masseter muscle, with loss of the separation border in places. Discreet filling of the two maxillary sinuses (Figure 2, Figure 3). Surgical exploration revealed a non-hemorrhagic gray mass filling the left maxillary sinus. An incisional biopsy was performed, and the histopathological analysis showed the presence of numerous histiocytes, some of which had evident emperipolesis (Figure 4).

**Diagnosis:** the final diagnosis retained is Rosai-Dorfman disease (MRD).

**Therapeutic interventions:** treatment offered by internal medicine doctors is corticotherapy; prednisone 5 mg.

**Figure 5:**
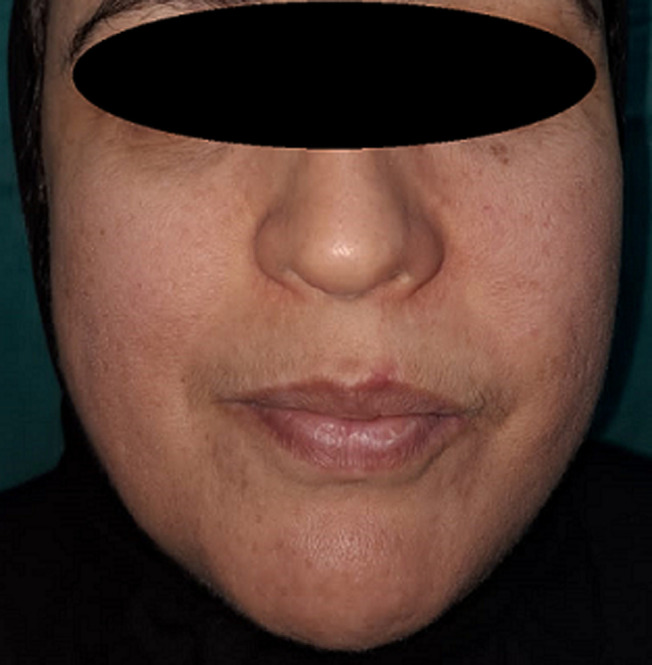
post-treatment appearance of Rosai-Dorfman patient after 1 year

**Follow-up and outcome of interventions:** the patient’s maxillary pain was controlled, the follow-up after 1 year of treatment showed an amelioration of the disease by corticotherapy (Figure 5).

**Patient perspective:** overall the patient’s experience with corticosteroid prednisone 5mg treatment was initially complex, causing so much discomfort and uncertainty, the response to the medication was promising, it has helped manage the symptoms and the swelling decreased.

**Informed consent:** the patient understands the nature of her condition, the proposed treatment options, potential risks and benefits. The patient gave informed consent.

## Discussion

In 1969, Rosai and Dorfman introduced a novel pathological entity distinguished by its unique clinical and histological features, predominantly manifesting within the first two decades of life [1,2]. Despite extensive epidemiological scrutiny, no discernible predilection for any particular race, gender, or socioeconomic class has been observed in cases of Rosai-Dorfman disease (RDD) [3,4]. Sinus histiocytosis with massive lymphadenopathy, commonly known as Rosai-Dorfman disease (RDD), represents a rare benign condition frequently characterized by painless bilateral cervical lymphadenopathy. Although extranodal involvement is uncommon, it can occur, affecting various regions such as the skin, nasal cavity, and paranasal sinuses. Such involvement may present with systemic symptoms including fever, pain, weight loss, pharyngitis, and nasal obstruction. Notably, osteolytic bone lesions are observed in a minority of cases, with only a small proportion affecting the jaw bones [5]. Among extranodal sites, involvement of the paranasal sinuses is notably common, with the maxillary region being one of the primary locations [6].

In our case, a woman of 42-years-old referred of extranodal RDD presenting as a progressively enlarging left maxillary intrabony mass. Computed tomography revealed a significant osteolytic lesion affecting the left maxilla, furthermore, both the physical examination and imaging indicated no other affected sites. The diagnosis of RDD was confirmed by histological and immunohistochemical studies. Macrophages exhibiting emperipolesis expressing S-100 and CD1a-negative-can be considered almost pathognomonic [7]. Differential diagnosis of bone lesions includes Langerhans cell histiocytosis (LCH), metastatic tumor, osteomyelitis, sarcoidosis and lymphoma. Malignant lymphoma can be excluded based on both clinical presentation and pathological findings. Furthermore, the absence of significant abnormal levels of lipids, carbohydrates, or mucopolysaccharides in the cytoplasm of histiocytes. The etiology of RDD remains uncertain; however, elevated antibody titers to human herpesvirus (HHV) 6 have been documented in patients with sinus histiocytosis with massive lymphadenopathy (SHML) or RDD [8].

There is no well-defined treatment protocol in the literature. Treatment can range from radiotherapy and chemotherapy in the event of severe manifestations with risk of compression, simple monitoring. According to Keskin *et al*. [9] surgery is the cornerstone and one of the most effective approaches. Conservative treatment should be considered in such cases, taking into account the possibility of a spontaneous remission. Overall, the prognosis for RDD is generally good, with most patients experiencing resolution of symptoms and long-term disease control with appropriate management. However, close monitoring and interdisciplinary management are essential to ensure optimal outcomes and timely intervention if complications arise.

## Conclusion

In our case of Rosie-Dorfman disease (RDD) localized in the maxillary region, the absence of lymph node involvement underscores the potential for extra-nodal manifestations, posing diagnostic challenges. The localized nature of the lesion suggests a more favorable prognosis; however, diligent monitoring for recurrence or progression remains crucial. This case emphasizes the importance of considering RDD's varied presentations and tailoring management strategies accordingly, advocating for close surveillance to detect any potential complications.
